# Design of an Intervention to Minimize Ingestion of Fecal Microbes by Young Children in Rural Zimbabwe

**DOI:** 10.1093/cid/civ845

**Published:** 2015-11-11

**Authors:** Mduduzi N. N. Mbuya, Naume V. Tavengwa, Rebecca J. Stoltzfus, Valerie Curtis, Gretel H. Pelto, Robert Ntozini, Rukundo A. Kambarami, Dadirai Fundira, Thokozile R. Malaba, Diana Maunze, Peter Morgan, Goldberg Mangwadu, Jean H. Humphrey

**Affiliations:** 1 Zvitambo Institute for Maternal and Child Health Research, Harare, Zimbabwe; 2 Division of Nutritional Sciences, Cornell University, Ithaca, New York; 3 Department of International Health, The Johns Hopkins Bloomberg School of Public Health, Baltimore, Maryland; 4 London School of Hygiene and Tropical Medicine, United Kingdom; 5 Aquamor Private Limited; 6 Ministry of Health and Child Care, Harare, Zimbabwe

**Keywords:** water, sanitation and hygiene, intervention design research, formative research, stunting, environmental enteric dysfunction

## Abstract

We sought to develop a water, sanitation, and hygiene (WASH) intervention to minimize fecal–oral transmission among children aged 0–18 months in the Sanitation Hygiene Infant Nutrition Efficacy (SHINE) trial. We undertook 4 phases of formative research, comprising in-depth interviews, focus group discussions, behavior trials, and a combination of observations and microbiological sampling methods. The resulting WASH intervention comprises material inputs and behavior change communication to promote stool disposal, handwashing with soap, water treatment, protected exploratory play, and hygienic infant feeding. Nurture and disgust were found to be key motivators, and are used as emotional triggers. The concept of a safe play space for young children was particularly novel, and families were eager to implement this after learning about the risks of unprotected exploratory play. An iterative process of formative research was essential to create a sequenced and integrated longitudinal intervention for a SHINE household as it expects (during pregnancy) and then cares for a new child.

Early childhood stunting, anemia, and diarrheal diseases are major causes of morbidity and mortality in the first 2 years of life. Recently, environmental enteric dysfunction (EED) has been hypothesized as a primary mediator of the association between fecally contaminated environments, stunting, and anemia [1–3]. EED is a subclinical condition of the small bowel characterized by reduced surface area [4] and increased permeability [5]. There is strong evidence linking EED with growth outcomes [5, 6], as well as observational evidence and plausibility [7, 8] that the pathogenesis of EED is related to environmental microbial contamination. However, it is not known whether improving water, sanitation, and hygiene (WASH) will prevent EED, or which microbes are important in EED etiology.

In the context of a cluster-randomized trial to reduce stunting and anemia by preventing EED (the Sanitation Hygiene Infant Nutrition Efficacy [SHINE] trial) [9], we sought to develop an intervention with high theoretical efficacy in minimizing ingestion of fecal microbes. Starting with evidence-based [10] WASH interventions (toilets, hand washing with soap, water treatment, and food safety), we undertook 4 phases of formative research to (1) contextualize conventional WASH interventions for rural Zimbabwe; (2) investigate the primary fecal–oral pathways of young children; (3) design interventions to interrupt these pathways; and (4) design a sequence of time-relevant behavior change communication lessons for delivery by community-based health workers to households during pregnancy and young childhood.

## Study Setting and Population

Formative research was conducted in the Sanitation Hygiene Infant Nutrition Efficacy (SHINE) trial districts (Chirumanzu and Shurugwi) and a neighboring district (Gweru) in central Zimbabwe. The livelihood of these communities is predominantly based on subsistence farming. Homesteads typically comprise a large yard where most household activities occur including child care and play, and separate huts for sleeping and cooking [11]; flooring is mainly earth and dung (41%) or cement (58%) [12]. Women almost exclusively assume primary responsibility for the care of young children [13], with younger siblings assisting with feeding and play. Literacy among women and men is 94% and 96%, respectively [12].

The Medical Research Council of Zimbabwe and the institutional review boards of Johns Hopkins University Bloomberg School of Public Health and the Research Institute of McGill University Health Centers approved these formative studies.

## METHODS AND RESULTS

### Phase 1: Contextualizing Conventional WASH Interventions for Rural Zimbabwe

The “F-diagram” [14, 15], which posits that microbes of fecal origin reach a new host through direct and indirect transmission pathways of fluids, fields, flies, fingers, or food, has been used for decades as the conceptual model of fecal–oral transmission. The objective of any WASH intervention is therefore to erect barriers that break the chain of enteric disease transmission from feces to a host. Consequently, WASH interventions are designed either as primary (eg, safe stool disposal and handwashing with soap after stool contact) or secondary (handwashing before cooking and eating, fly control, and drinking water treatment) [10] barriers to these “F” pathways.

#### Methods

To obtain an emic perspective of current WASH practices [16], we conducted 4-hour structured observations and qualitative interviews [17] in 21 households with children aged 1–18 months. We recorded living conditions, behaviors around the disposal of feces, handwashing, and water usage. In-depth interviews were conducted with the mother and other adults regarding water, hygiene, and sanitation practices [17]. Text data were analyzed thematically, to identify frequently stated problems [18].

Behavior microtrials [17] were completed among 19 of the study households following the Designing by Dialogue approach [18]: Mothers were counseled about the identified problems and invited to choose and try 1 or more improved practices. Households were visited twice over the subsequent 2 weeks to determine uptake of chosen practices and renegotiate if necessary [18]. One year later, 15 participants were visited to ascertain long-term maintenance of the negotiated behaviors. We also conducted key informant interviews and held focus group discussions with community members to augment findings from the household study.

#### Findings

##### Human Feces Disposal

Participants valued toilet ownership, particularly for disease prevention, and expressed feelings of disgust associated with visible feces “all over the place,” and loss of dignity associated with open defecation:

You can be disturbed while in the process of defecating  …  if caught you get ashamed, lose respect  …

Of the 21 households, 5 (24%) had a latrine and another 10 households (48%) either had one in the recent past, used a neighbor's latrine, or planned to build one but lacked resources (especially cement) required for a ventilated improved pit (VIP) latrine. Fifteen of 16 households (94%) without latrines spontaneously (without prompt) said they would like one. Thus, in our small sample, the desire and demand for adequate sanitation were high.

Based on household interviews and focus group discussions, people commonly believed that child feces are safer, less infectious, and less disgusting than adult feces:

Once baby feces dissolve in water, it is not very infectious. One can just throw the dirty water in the open.

##### Handwashing

During 84 hours of household observation, we observed 93 opportunities for handwashing. Of these, 33 (35%) included handwashing with water only and 6 (6%) with soap and water. Within 2 weeks of counseling, all study households had built and were using a Tippy Tap, a simple device (in these trials, using a discarded plastic bottle) that ensures a thin stream of water, suitable for economical handwashing. After 1 year, 12 of the 15 (80%) households still had a Tippy Tap installed, with evidence of use (water in the container and on the ground around the device).

##### Animal Feces Disposal

Animal feces were visible in all homesteads, and uncorralled domestic animals (mostly chickens) had access to living areas. None of the households chose to try corralling chickens even when the practice was renegotiated on the first follow-up visit. The reason given was economic: Corralled chickens would be unable to forage for food and would therefore need to be fed and treated for parasites—a perceived risk associated with corralled poultry. This reason was confirmed in subsequent focus group discussions.

##### Water Quality

Only 6 of 21 households had access to a protected drinking water source. Participants commonly believed they could assess water contamination visually. Five of 21 households reported purifying their drinking water. All 16 households were offered a trial behavior, either boiling all drinking water for the family or boiling the water given to the young child. Ten of the remaining 16 families agreed to try boiling water for drinking; all reported having done this for the 2-week trial period and, 1 year later, 11 were still boiling their drinking water. Water storage containers were uncovered in 10 of 19 households; all 10 began covering the container over the 2-week trial and, 1 year later, all reported continuing this practice.

From analysis of the individual interviews and focus group discussion transcripts, we identified 2 different emotions to motivate WASH behaviors: disgust and nurture. Visible feces and physical contact with feces elicit disgust. Also, parents aspired to have healthy, intelligent children. Highlighting that children can touch and ingest feces in the yard triggers both disgust and nurture, emotions that drive hygiene behavior [19–23].

### Phase 2: Targeting Interventions to the Developing Young Child

While the F-diagram describes primary fecal–oral transmission pathways, it may not be valid for infants whose primary food and fluid is breast milk and who regularly mouth objects as part of their normal development. In rural areas of developing countries, young children crawl and play in environments laden with microbes, not just those of human fecal origin. These insights motivated phase 2, in which we investigated the microbe transmission pathways potentially most relevant for children in the SHINE target age range of 0–18 months. Detailed methods and results from this phase have been published elsewhere [24].

By starting with the behavioral endpoint (fecal microbes entering the infant's mouth) and working backward, we identified 3 pathways of microbial ingestion that were not adequately addressed by conventional WASH interventions: contact with soil, contact with chicken feces, and the child's own hands [25]. Crawling on bare contaminated soil and kitchen floors exposes young children to animal and human fecal bacteria. Washing crawling infants' hands with soap would have to be practiced with implausible regularity to prevent hand-to-mouth microbe transmission. And given the uncertain etiology of EED, it is possible that we also need to be concerned with a broader range of microbes than human fecal–oral diarrheal pathogens. We concluded that these are additional pathways that must be interrupted to prevent EED within the first 2 years of life.

### Phase 3: Developing Materials and Messages for a Safe Play Space

Realizing that ingested microbe consumption from hand-to-mouth behaviors dwarfed exposure from food and water [24], we were compelled to expand our WASH intervention package to protect babies from contaminated soil and chicken feces. Given phase 1 findings that corralling chickens was economically unacceptable in this setting, and because toilet provision was already a component of the trial intervention, we decided to design a safe play space as part of our intervention package for crawling and toddling children.

#### Methods

We developed messages to inform parents that eating soil and chicken feces was bad for their child's health and to promote having children play on a washable mat, especially when eating. We pilot-tested these messages among 20 rural households with children aged 0–18 months in Gweru rural district. We explored the acceptability of commercial play yards using pictures and 4 models imported from the United States.

We conducted focus group discussions with participants before and after exposure to the behavior change messages. Discussions were tape recorded, transcribed, and analyzed thematically.

#### Findings

In initial focus group discussions and structured interviews, parents confirmed that soil and chicken feces ingestion by children is common and that elders advised that eating soil was good for the baby and treated stomach ailments. Parents were resistant to confining their children to a protective area. However, after they were exposed to the messages highlighting the health risks associated with ingesting soil and chicken feces, parents became more receptive:

From what our elders had taught that eating soil makes the baby's stomach strong or can treat *nhova* [depressed fontanelle] and the baby will not fall sick often. I have changed on that.

I used to know that babies should not eat chicken feces because that child would bite other babies, and also that soil helps to heal the baby's stomach pains. So to me in the past it did not matter  …  but now because of the lesson I know that soil contains chicken feces that can cause diarrhea.

Notably, the diarrhea risk perception was influenced also by the 2008–2009 cholera epidemic in Zimbabwe [26], which coincided with our study.

After understanding the health risks associated with ingesting soil and chicken feces, all mothers understood the value of using a mat and a protective play space:

I can see that it is a good thing and it can make my child not go out and play with the soil and eat it. Also the baby will be entertained in it  …  I can see that even if the child wants to stand, he can still use its walls and walk around.

Regarding various models of play yards, parents were most attracted to sturdy, brightly colored plastic models that allowed the child to move about and pull himself to standing positions. They also favored portable models that could be moved about the homestead or taken to the field.

### Phase 4: Designing a Sequenced Intervention to Minimize Fecal Ingestion by Young Children

Based on learning about local sanitation hardware policy, current WASH knowledge, attitudes, and practices among households, and potential pathways of fecal–oral transmission among young children, our final intervention to minimize infant fecal microbe ingestion included:

- Provision of a Blair VIP latrine, 2 handwashing facilities (Supplementary Appendix 1.3), regularly (ie, monthly) replenished liquid soap, a washable play mat and play yard, and WaterGuard point-of-use chlorination from 5 months postpartum, in preparation for the end of exclusive breastfeeding and start of giving drinking water to infants.
- Five behavior change modules (presented in Supplementary Appendices 1.1 and 1.2), grounded in behavior change theory [20, 27] and designed to invoke motivating emotions for hygiene (disgust and nurture), each delivering 1 key message:
  1. Safely dispose of all animal and human feces;
  2. Wash hands with soap after fecal contact and before preparing food, eating food, or feeding children;
  3. Protect children from ingesting soil and animal feces;
  4. Freshly prepare children's food, or reheat to boiling prior to feeding;
  5. Give children (after 6 months of exclusive breastfeeding) only drinking water that has been chlorinated.

Delivery of hardware, commodities, and modules were sequenced longitudinally to successively review and build on previous messages and deliver new messages at the most relevant age of the fetus/infant. Intervention components and timing are detailed in Table 1. By sequencing and timing the messages, we intended to avoid message overload, to tap into the strong emotional trigger around nurture of a new child, and to deliver messages about baby care at the most relevant (teachable) moments for the mother and other caregivers [28].

**Table 1.** Behavior Change Intervention Messages and Material Inputs to Interrupt Vectors of Microbial Transmission Among Rural Zimbabwean Children 0–18 Months of Age

Timing of Intervention	Source or Vector	Material Inputs	Key Behavioral Message	Psychological Trigger
25–29 wk gestation	Feces (human and animal)	Ventilated improved pit latrine	Safely dispose of all animal and human feces. Keep yard swept of all types of feces. Discard feces, nappy water, animal feces not used for fertilizer in the latrine. Compost animal feces for fertilizer in a pit away from play area of children. Keep latrine clean and available	Disgust, nurture
29–33 wk gestation	Hands (caregivers and child)	2 Tippy Taps and liquid soap	Wash hands with soap after fecal contact, before preparing food, eating food, or feeding the child. Use and maintain Tippy Taps. Wash baby's hands after nappy change, when visibly dirty, and routinely 3–4 times throughout the day.	Disgust, nurture
8–12 wk postpartum	Soil, chicken feces	Baby mat^a^ Play yard^b^	Keep your child from eating soil and chicken feces. Put your child in a clean protected play space when playing or eating, where he/she cannot access contaminated soil and chicken feces. Once baby is mobile, use play yard in addition to play mat.	Nurture
16–20 wk postpartum	Water	Point-of-use chlorination agent (WaterGuard^c^)	Treat all children's drinking water with WaterGuard. Store drinking water in a covered container and use hygienic methods for extracting water from the storage container. Always give your baby water treated with WaterGuard after 6 mo of age.	Nurture
21–25 wk postpartum	Food	None	Freshly prepare or reheat to boiling all children's food. Wash foods that can be eaten raw. Wash eating utensils with soap and water. As much as possible, freshly prepare small portions of food for your child. Store leftovers in clean containers with tight-fitting lids. Reheat leftovers by bringing to a boil. Discard leftovers that have been reheated once.	Nurture

^a^ Mat is 2.8 × 3.0 m, manufactured locally from plastic weave material.

^b^ North States, Minneapolis, Minnesota.

^c^ Dilute sodium hypochlorite solution (manufactured and distributed locally by Nelspot, Zimbabwe).

#### Latrine Construction

The process of latrine construction has been the most technically and logistically demanding component of the WASH intervention. The Blair VIP latrine was designed in Zimbabwe in the 1970s [29] and remains the most commonly exported public health innovation. Until 2013, and in line with this legacy, it was the approved sanitation facility for rural areas. Blair VIPs provide sanitation to a 6-member family for 12–15 years, and reduce foul odor and fly populations in the homestead. Since 2012 when the SHINE trial began, policy and programming has expanded to promote construction of “upgradable” VIPs: fully lined pits with sealed cement slabs with temporary superstructures made of grass, plastic, or other material. As families can afford, they are encouraged to upgrade the superstructure using bricks and cement, including a ventilation mechanism.

After the first 300 units or so were constructed within WASH households, Dr Peter Morgan, the inventor of the original Blair VIP, designed a new model that reduced implementation cost and increased efficiency (using 40% less brick and cement materials), and had a wider but shallower pit designed to be more stable in the sandy soils of our study districts (details available at www.aquamor.info). The government of Zimbabwe approved this model. We elected to fully subsidize the cost of the latrine to avoid any heterogeneity in latrine construction. Our formative work confirmed cost as the main barrier to latrine ownership in this context and reassured us that latrine ownership is highly valued.

Latrines were constructed at households of women enrolled into the WASH or WASH + IYCF clusters, optimally within 6 weeks of recruitment or <26 weeks' gestation; with the allowable window closing at time of parturition. Early failure by a subcontracted organization to meet these requirements of the trial, resulting in only 9% of the 737 women enrolled into WASH arms between November 2012 and July 2013 having received a latrine, triggered a change in implementation approach. From September 2013, latrine construction was implemented by the Environmental Health Department of the Ministry of Health and Child Care (EH-MoHCC), with management and logistical support from Zvitambo. Teams comprising 23 environmental health technicians, approximately 200 local excavators, and approximately 170 builders were mobilized from the 2 districts, and trained on the modified Blair VIP. Standard operating procedures were developed and tracked in a computerized database to document and monitor progress on latrine construction in each WASH household. In brief, following enrollment of a woman into a WASH arm, an environmental health technician visited the household and sited a safe location for a new latrine in consultation with the family, engaged an excavator to dig the pit, ordered delivery of building supplies from the SHINE hub within a week, and assigned a trained builder to complete the latrine. A small proportion of households (<5%) had an existing latrine that met EH-MoHCC standards and was less than one-half full, and therefore did not need a new latrine constructed.

## DISCUSSION

The WASH intervention was designed to achieve the specific goal of minimizing the ingestion of fecal microbes by infants and young children. Our focus on babies was essential in the context of the SHINE trial primary aims of improving length and hemoglobin concentration (or reducing stunting and anemia, respectively) at 18 months [9]. Given the importance of early childhood stunting, anemia, and diarrheal morbidity and mortality in the first 1000 days of life—a critical window of opportunity for child growth and development—a “baby-focused” approach to WASH programming may be relevant to many global nutrition and health initiatives.

We designed behavior change interventions to elicit disgust and nurture emotions, which are strong promoters of hygiene behavior [19–23] and are already salient within Zimbabwean culture. We did not pursue the use of shame as an emotional trigger, despite its prominence in some sanitation interventions, because of the well-documented evidence linking shame to antisocial behaviors [30, 31]. These WASH modules are based on evidence-based principles of adult education [32], and include stories, images, demonstrations, and other participatory activities. Although the primary interaction is between the village health worker (VHW) and the mother, VHWs invite other household members to participate. All VHWs were trained to interact with respect, to frequently probe for questions, to express empathy with the challenges the mother is facing, and to praise positive changes and behaviors.

A novel part of our intervention is provision of a protective mat and play yard (illustrated in Supplementary Appendix 1.3). We are not the first to recognize the hazards of exploratory hand-to-mouth behaviors in the crawling and toddling child in contexts of poverty, nor the first to highlight the particular hazard of chicken feces. A study in Lima, Peru, reported 3.9 (standard deviation, 4.6) episodes of ingestion of chicken feces by children aged <5 years who underwent similar spot observations over a 12-hour period [33]. A recent comprehensive review also reported that human geophagia (consumption of earth) is common among children in low-income countries, where pathogen densities are highest [34]. Also, caregiver-reported geophagy was recently shown to be significantly associated with markers of intestinal inflammation and greater odds of Bangladeshi children being stunted [35]. The protective play space could be a key component of baby-focused WASH. Our formative research focused only on acceptability, however, and the longer-term uptake, effects, and potential risks of this intervention are not yet known.

Within SHINE, the WASH intervention is delivered to individual households, rather than to whole communities. Some have argued that total community-level sanitation is necessary to achieve impact on health outcomes [36]. Our strategy deviates from that approach, for several reasons. First, SHINE is a cluster-randomized trial with clusters defined by VHW catchment area, and these clusters do not necessarily correspond to social communities. Second, homesteads in these rural districts are not always clustered in large compounds or villages, as is typical of other contexts. Often they are strikingly isolated. Third, the cost of providing community-level sanitation to whole VHW catchment areas, including households without pregnant women, was prohibitive. Recent findings from Bangladesh comparing EED and stunting in individual households with best and worst WASH provide assurance that household-level cleanliness may be most relevant to infant health [37].

Water is the least prominent aspect of our overall package, consisting only of provision and education about point-of-use water chlorination. Water access is problematic throughout the SHINE study area and there is virtually no piped water. We explored water access interventions, such as drilling boreholes in the worst-off locales or promoting the use of water transport devices such as Hippo Water Rollers (Hippo Water Roller Project, South Africa). However, we chose not to intervene on water access, and instead opted to constrain our randomization on water access, which was assessed in a pretrial water point survey [38]. At baseline and all postnatal visits, a questionnaire module ascertains household water access: source, type, walking time, distance of water for drinking and water for uses other than drinking, and 24-hour recall of household water collection. The local name of the water point is elicited to link the household water source to the water point survey data. Therefore, our trial is designed to make inference about the effects of the WASH intervention package in an unbiased manner with regard to internal validity, while also measuring the modifying effect of water access [39] to inform how our results might be generalized to other settings with different water access.

A major reason the SHINE WASH intervention can be implemented feasibly and acceptably is that it is delivered by district- and community-level providers in the government sector, with strong local ownership. It is important to note the high rate of literacy throughout Zimbabwe, and the high degree of training and professionalism of (institution- and community-based) government staff.

## CONCLUSIONS

The principles guiding development of this intervention were to clearly identify behaviors that cause risk, to use qualitative research to understand why people engage in these behaviors, to trace the microbiology through the vectors, and to combine creative thinking with theory. The resulting package is tailored to context with strong active ingredients, such as emotions, suitable products to change the environment where needed, and presented in a format that engages adult learners.

## Supplementary Data

Supplementary materials are available at *Clinical Infectious Diseases* online (http://cid.oxfordjournals.org). Supplementary materials consist of data provided by the author that are published to benefit the reader. The posted materials are not copyedited. The contents of all supplementary data are the sole responsibility of the authors. Questions or messages regarding errors should be addressed to the author.
